# Occupational Violence, Organisational Factors, Burnout and Workforce Withdrawal Intentions Among Inpatient Mental Health Staff: A Cross-Sectional Survey

**DOI:** 10.3390/healthcare14193158

**Published:** 2026-09-23

**Authors:** Mohamed Jalloh, Samukeliso Fundira, Humberto Gomez, Mason Jones, Maryam Nusrat Javed, Vasudevan Krishnan

**Affiliations:** 1 Royal Stoke University Hospital, University Hospitals of North Midlands, Stoke-On-Trent ST4 6QG, UK; samukeliso.fundira@uhnm.nhs.uk (S.F.); humberto.gomez@uhnm.nhs.uk (H.G.); maryam.javed@uhnm.nhs.uk (M.N.J.); 2 St George’s Hospital, Midlands Partnership University NHS Foundation Trust, Stafford ST16 3SR, UK; vasudevan.krishnan@mpft.nhs.uk

**Keywords:** burnout, healthcare workforce, inpatient mental health, mental health staff, occupational violence, staff wellbeing, workforce retention

## Abstract

**Aim/Objective:** To examine associations between occupational violence, organisational factors, burnout, and workforce withdrawal intentions among inpatient mental health staff. **Method:** An anonymous cross-sectional survey of 115 inpatient mental health staff within a United Kingdom mental health trust was conducted. Burnout was assessed using a seven-item composite derived from the Copenhagen Burnout Inventory. Multivariable linear and ordinal logistic regression examined associations with burnout and workforce withdrawal intentions, respectively. **Results:** Verbal aggression was reported by 93.9% of respondents, including 41.7% at least weekly. Burnout differed across verbal-aggression frequency categories (*F*(5,109) = 2.43, *p* = 0.040). Lower perceived post-incident support (standardised β = −0.32; *p* = 0.001) and staffing adequacy (standardised β = −0.27; *p* = 0.015) were independently associated with higher burnout, whereas verbal aggression was not (*p* = 0.191). Higher burnout was associated with greater intention to leave the current role (aOR = 11.54, 95% CI 5.96–22.34; *p* < 0.001) and greater intention to reduce working hours (aOR = 3.49, 95% CI 2.16–5.66; *p* < 0.001). **Conclusions:** Organisational conditions, particularly perceived post-incident support, were associated with burnout after accounting for occupational violence exposure and staff characteristics. The cross-sectional design precludes causal inference, while the single-service setting and modest sample size may limit generalisability. Staffing adequacy and post-incident support represent potentially modifiable organisational factors warranting further investigation in relation to staff wellbeing and workforce sustainability. Longitudinal and interventional studies should evaluate whether changes in organisational conditions are associated with subsequent changes in burnout and workforce withdrawal intentions.

## 1. Introduction

Burnout among staff working in inpatient mental health services is an important occupational health and workforce concern, with implications for staff wellbeing, workforce sustainability, and the quality and safety of patient care [1,2]. Staff in these settings manage complex mental illness, behavioural disturbance, and high clinical acuity, often alongside recurrent occupational violence [3,4,5]. Verbal aggression, threats, physical assault, and other hostile or challenging behaviours are frequently encountered, and non-physical aggression may also be associated with psychological strain and burnout [6,7,8,9].

Burnout has been associated with adverse occupational outcomes, including greater intention to leave and workforce turnover [10,11,12]. However, occupational violence occurs within a wider organisational environment. Staffing, managerial and peer support, leadership, and the practice environment have also been associated with healthcare-worker wellbeing and burnout [13,14,15]. Recent evidence among inpatient psychiatric nurses similarly indicates associations between more favourable work environments and lower incidence of burnout, job dissatisfaction, and intention to leave [16].

These relationships can be considered within the Job Demands–Resources (JD-R) model, which proposes that occupational wellbeing reflects the balance between work demands requiring sustained effort and resources that support employees in meeting those demands [14,17]. In inpatient mental health services, recurrent aggression may represent an occupational demand, while adequate staffing and effective organisational and post-incident support may represent workplace resources. The JD-R model therefore provides a rationale for examining occupational violence and organisational conditions concurrently. Given the cross-sectional design, it is used here as a conceptual framework rather than to imply a causal pathway.

Recent mental health nursing research supports consideration of these domains together. Park et al. identified relationships between violence exposure, social support, the nursing practice environment, burnout, and mental health among mental health nurses, while Dixon et al. highlighted associations between the work environment, burnout, and intention to leave among inpatient psychiatric nurses [16,18]. In the wider UK healthcare workforce, Collett et al. found that perceived workplace support, including staffing and safety, managerial support, leadership, and collegial support, was associated with burnout and mental wellbeing [15].

Despite this evidence, relatively limited research has examined occupational violence and potentially modifiable organisational factors concurrently in relation to burnout among inpatient mental health staff while also considering workforce withdrawal intentions. Existing studies have also frequently focused on particular professional groups, especially nurses, despite inpatient mental health care being delivered by multidisciplinary teams. Examining these factors together across professional groups may therefore provide further insight into their relationships within inpatient mental health services.

Accordingly, this study examined associations between occupational violence exposure, perceived organisational factors, burnout, and workforce withdrawal intentions among inpatient mental health staff. Three research questions were addressed: (RQ1) What are the associations between occupational violence exposure, perceived organisational factors, and burnout among inpatient mental health staff? (RQ2) Are perceived staffing adequacy and post-incident support associated with burnout after accounting for occupational violence exposure and staff characteristics? (RQ3) Is higher burnout associated with greater intention to leave the current role and greater intention to reduce working hours?

## 2. Materials and Methods

### 2.1. Ethics, Governance and Consent

This study was registered locally as a quality improvement project/service evaluation within Midlands Partnership University NHS Foundation Trust (MPFT) [ID: 276]. An anonymous cross-sectional staff survey was undertaken under local institutional governance arrangements. In accordance with UK Health Research Authority guidance, NHS Research Ethics Committee review was not required because the project was conducted as a service evaluation using anonymised staff survey data without collection of patient information or identifiable participant data.

Participation was voluntary. Before accessing the questionnaire, participants received information outlining the survey purpose, anonymity, intended use of the data including academic presentation and publication, and their right to skip questions or discontinue participation. Completion and submission of the anonymous questionnaire constituted informed consent.

### 2.2. Study Design and Participants

A cross-sectional anonymous survey was conducted between 5 January and 1 February 2026 among clinical staff working within inpatient mental health services at MPFT. Recruitment was centred on St George’s Hospital, Stafford. Of the final sample, 112 participants (97.4%) reported St George’s Hospital as their primary site of work, while three (2.6%) specified Redwoods Centre, Shrewsbury.

Clinical staff working within psychiatric inpatient services during data collection were eligible, including doctors, nurses, healthcare assistants/support workers and allied health professionals. Respondents were excluded if they selected “No” in response to the mandatory initial consent/eligibility question; no additional exclusion criteria based on professional group, duration of employment or work pattern were applied.

Participants were recruited using non-probability convenience sampling. The electronic questionnaire was distributed through a staff email circulated by the clinical supervisor and QR-code posters displayed on participating inpatient wards, with one reminder email approximately two weeks before survey closure. Participation was voluntary, and no incentives were offered.

A total of 116 responses were received. One respondent selected “No” to the mandatory consent/eligibility question and was excluded, leaving 115 participants (Figure 1). As the total number of eligible staff who received or viewed the invitation could not be reliably determined, a conventional response rate could not be calculated. No a priori representative sample-size calculation was undertaken because this was a service evaluation using voluntary recruitment over a predefined data-collection period; the achieved sample comprised eligible staff who participated during this period.

### 2.3. Survey Design and Data Collection

The anonymous questionnaire collected professional and occupational characteristics, occupational violence exposure, organisational conditions, work-related burnout and workforce withdrawal intentions. Participant characteristics included professional role, primary site, work setting, years working in psychiatry and work pattern. Age, sex/gender, clinical grade, typical weekly working hours, night-shift frequency and specific shift pattern were not collected.

Occupational violence during the preceding six weeks was assessed using study-specific frequency items covering verbal aggression, threats of physical harm, attempted assault, physical assault, sexually inappropriate or sexually harassing behaviour, and discriminatory or racist abuse, with responses ranging from no exposure to daily or almost daily exposure. Sexually inappropriate or harassing behaviour referred specifically to behaviour from patients, including sexual comments, gestures and unwanted exposure. Additional questions assessed involvement in physical restraint, seclusion and rapid tranquillisation.

Organisational conditions were assessed using study-specific single items covering perceived physical safety, training adequacy for managing aggression, post-incident support and processes, incident reporting and staffing adequacy. Responses used five-point Likert scales from strongly disagree to strongly agree, with higher scores indicating more favourable perceptions. Workforce withdrawal intentions were assessed using study-specific questions asking how likely participants were, within the following 12 months, to leave their current role or reduce working hours because of work-related stressors, using five ordered categories from very unlikely to very likely. These study-specific measures were developed for the service evaluation and had not undergone formal psychometric validation.

### 2.4. Burnout Assessment

Work-related burnout was assessed using the seven-item work-related burnout subscale of the Copenhagen Burnout Inventory (CBI) [19]. Responses were coded from 0 to 4, with the positively worded item assessing energy for family and friends reverse-scored so that higher values consistently indicated greater burnout. The composite score was calculated as the mean of the seven items, giving a possible range of 0–4; multiplying this score by 25 yields the conventional 0–100 CBI metric. The 0–4 metric was retained for analysis, and complete burnout-item data were available for all 115 participants.

Internal consistency was assessed using Cronbach’s alpha, corrected item–total correlations and alpha if each item was deleted. Internal consistency was high (Cronbach’s α = 0.915); full item-level reliability statistics are provided in Supplementary Table S1.

### 2.5. Outcomes

The primary outcome was work-related burnout, analysed as a continuous composite score. Secondary outcomes were intention to leave the current role and intention to reduce working hours within the following 12 months because of work-related stressors, with each measured as a five-category ordinal outcome.

### 2.6. Statistical Analysis

Categorical variables were summarised using frequencies and percentages, and continuous variables and composite scores using means and standard deviations (SD) where appropriate. Burnout-score distribution was examined using histograms, quantile–quantile plots and the Shapiro–Wilk test.

Spearman’s rank correlations examined bivariate associations between burnout, occupational violence, organisational variables and workforce withdrawal intentions. One-way analysis of variance (ANOVA) compared mean burnout across verbal-aggression frequency categories; the omnibus ANOVA was interpreted as evidence of differences between categories rather than a progressive dose–response relationship.

Multivariable linear regression examined adjusted associations with burnout. The primary model included verbal aggression, threats of physical harm and physical assault as the principal occupational violence exposures; perceived staffing adequacy and post-incident support as the principal organisational variables; and professional role group, years working in psychiatry and work pattern as staff-level covariates. Violence exposures were selected a priori on clinical grounds as commonly encountered forms of inpatient occupational violence, while staffing adequacy and post-incident support were selected as service-relevant and potentially modifiable organisational conditions. Predictor inclusion reflected the clinical and organisational focus of the analysis rather than automated statistical selection. Professional role was categorical, with medical staff as the reference group. Unstandardised coefficients (B), 95% confidence intervals (CIs), standardised beta coefficients (β) and *p*-values were reported.

Regression assumptions were assessed using residual and quantile–quantile plots, the Shapiro–Wilk test, the Breusch–Pagan test and variance inflation factors. Influence and stability were assessed using Cook’s distance, leverage, studentised residuals and DFBETAs, with HC3 heteroscedasticity-consistent standard errors and leave-one-out analyses used as robustness checks.

As a sensitivity analysis, the primary model was expanded to include perceived physical safety and training adequacy. Primary and expanded models were compared on the same complete-case sample using model-fit statistics and nested-model comparison, with regression assumptions and HC3 robust inference also assessed for the expanded model.

An exploratory hierarchical regression examined incremental variance in burnout. Staff-level characteristics were entered first, followed by occupational violence exposures and organisational variables, reflecting the study’s conceptual framework rather than statistical significance. Changes in model fit were assessed using R^2^, adjusted R^2^, change in R^2^ and F-change statistics.

The five-category workforce withdrawal outcomes were analysed using cumulative-link ordinal logistic regression, with burnout as the principal explanatory variable and adjustment for professional role, years working in psychiatry and work pattern. The proportional-odds assumption was assessed using nominal-effects tests. For intention to leave, non-proportional odds were identified for years working in psychiatry, so a partial proportional-odds model allowed its effect to vary across thresholds while retaining proportional effects for the remaining predictors. The assumption was satisfied for intention to reduce working hours. Associations are reported as adjusted odds ratios (aORs) with 95% CIs and *p*-values.

Exploratory subgroup analyses examined differences in burnout and occupational violence exposure across professional role groups using one-way ANOVA or categorical tests as appropriate.

Complete-case data were used, and missing values were not imputed. Four participants (3.5%) with missing post-incident support data were excluded from the primary burnout model, giving *n* = 111; no other primary-model variables had missing data. Missingness was explored by comparing included and excluded participants across available characteristics, violence exposures and burnout using Wilcoxon rank-sum and Fisher’s exact tests as appropriate. No statistically significant differences were identified, although these comparisons were limited by the four excluded participants. All 115 participants had complete data for the workforce withdrawal models.

All tests were two-sided, with *p* < 0.05 considered statistically significant. Analyses were performed using R version 4.5.0. Reporting followed the Strengthening the Reporting of Observational Studies in Epidemiology (STROBE) guidelines for cross-sectional studies.

## 3. Results

### 3.1. Participant Characteristics and Occupational Violence Exposure

A total of 116 responses were received. One respondent did not confirm the mandatory consent/eligibility question and was excluded, leaving 115 participants (Figure 1). Participant characteristics, occupational violence exposure, organisational perceptions and burnout scores are summarised in Table 1. Most respondents reported St George’s Hospital as their primary site (112/115, 97.4%); three (2.6%) reported Redwoods Centre. Adult acute (39.1%) and forensic wards (26.1%) were the most common settings, while registered mental health nurses (33.9%) and healthcare assistants/support workers (23.5%) were the largest professional groups. Most respondents worked full-time (84.3%), and 44.3% had more than five years’ experience in psychiatry.

Occupational violence during the preceding six weeks was common. Verbal aggression was reported by 93.9% of respondents, including 41.7% at least weekly. Threats of physical harm were reported by 73.9%, physical assault by 58.3%, and sexually inappropriate or sexually harassing behaviour from patients by 72.2% (Table 1). Detailed frequency distributions are presented in Supplementary Table S2.

Mean organisational scores were 2.29 (SD 1.09) for staffing adequacy, 2.64 (SD 0.99) for post-incident support, 2.68 (SD 0.92) for physical safety and 2.70 (SD 0.95) for training adequacy. Mean burnout was 1.92 (SD 0.87) (Table 1).

### 3.2. Occupational Violence and Burnout

Burnout differed significantly across verbal-aggression frequency categories (*F*(5,109) = 2.43, *p* = 0.040, η^2^ = 0.10) (Supplementary Table S3; Supplementary Figure S1). Homogeneity of variance was supported by Levene’s test (*p* = 0.370), and Welch ANOVA yielded a similar result (*F*(5,27.27) = 3.09, *p* = 0.025). Tukey comparisons identified higher burnout among staff reporting verbal aggression daily or almost daily than among those reporting none (mean difference = 1.37, adjusted *p* = 0.017); no other pairwise comparisons were significant. The findings therefore indicate differences between exposure categories rather than a progressive dose–response relationship.

Burnout was weakly positively correlated with verbal-aggression frequency (Spearman’s ρ = 0.20, *p* = 0.031), while associations with threats of physical harm (ρ = 0.16, *p* = 0.081) and physical assault (ρ = 0.06, *p* = 0.495) were not significant. Higher burnout was associated with lower perceived staffing adequacy (ρ = −0.42, *p* < 0.001), lower post-incident support (ρ = −0.41, *p* < 0.001), greater intention to leave (ρ = 0.66, *p* < 0.001), and greater intention to reduce working hours (ρ = 0.45, *p* < 0.001) (Supplementary Table S4).

### 3.3. Multivariable Associations with Burnout

The primary multivariable model included 111 participants with complete data (Table 2; Supplementary Figure S2). Four participants (3.5%) were excluded because of missing post-incident support data; no other primary-model variables were missing. No statistically significant differences were identified between included and excluded participants across the available characteristics examined, although comparisons were limited by the small number excluded.

Lower perceived post-incident support (B = −0.278, 95% CI −0.442 to −0.113; β = −0.32; *p* = 0.001) and staffing adequacy (B = −0.213, 95% CI −0.383 to −0.042; β = −0.27; *p* = 0.015) were independently associated with higher burnout. Verbal aggression was not independently associated with burnout after adjustment (B = 0.116, 95% CI −0.058 to 0.290; β = 0.17; *p* = 0.191), nor were threats of physical harm (B = 0.026, 95% CI −0.174 to 0.226; *p* = 0.798) or physical assault (B = −0.105, 95% CI −0.338 to 0.128; *p* = 0.374). Years working in psychiatry and work pattern were also not significant.

The overall model was significant (*F*(10,100) = 3.72, *p* < 0.001; R^2^ = 0.271; adjusted R^2^ = 0.198). Diagnostics indicated no problematic multicollinearity (adjusted GVIFs 1.06–1.71), residual non-normality (Shapiro–Wilk *p* = 0.949), or heteroscedasticity (Breusch–Pagan *p* = 0.899). The burnout-score distribution is shown in Supplementary Figure S3. HC3 robust standard errors produced the same substantive findings, with staffing adequacy (*p* = 0.035) and post-incident support (*p* = 0.007) remaining significant.

Hierarchical regression showed that staff-level characteristics explained little variation in burnout (R^2^ = 0.025; adjusted R^2^ = −0.021). Occupational violence exposures added 3.6% but did not significantly improve model fit (ΔR^2^ = 0.036; Δ*F* = 1.67; *p* = 0.179), whereas staffing adequacy and post-incident support added 20.9% (ΔR^2^ = 0.209; Δ*F* = 14.35; *p* < 0.001), producing a final R^2^ of 0.271 (adjusted R^2^ = 0.198) (Supplementary Table S5).

Exploratory analyses found no differences in burnout across professional role groups (*F*(3,111) = 0.42, *p* = 0.737, η^2^ = 0.01). Verbal-aggression frequency differed overall between groups (*F*(3,111) = 3.23, *p* = 0.025, η^2^ = 0.08), although no Tukey-adjusted pairwise comparison was significant (Supplementary Table S6).

### 3.4. Robustness

Leave-one-out analyses showed that post-incident support remained significant in all 111 models and staffing adequacy in 110/111; both associations remained negative throughout. Occupational violence variables remained non-significant in all models.

In the sensitivity model additionally including physical safety and training adequacy, post-incident support remained independently associated with burnout (B = −0.222, 95% CI −0.399 to −0.044; *p* = 0.015), while staffing adequacy was attenuated (B = −0.151, 95% CI −0.336 to 0.034; *p* = 0.108). Neither physical safety (*p* = 0.174) nor training adequacy (*p* = 0.729) was independently associated with burnout, and their addition did not significantly improve model fit (*p* = 0.235).

### 3.5. Burnout and Workforce Withdrawal Intentions

In adjusted ordinal regression, higher burnout was strongly associated with greater intention to leave the current role (Table 3). Because the proportional-odds assumption was not satisfied for years working in psychiatry (*p* = 0.044), a partial proportional-odds model allowed its effect to vary across thresholds. Each one-unit increase in burnout was associated with 11.54-fold higher adjusted odds of being in a higher leave-intention category (aOR = 11.54, 95% CI 5.96–22.34; *p* < 0.001), with no remaining evidence of non-proportionality among predictors constrained to proportional effects.

Higher burnout was also associated with greater intention to reduce working hours. The proportional-odds assumption was satisfied, and each one-unit increase in burnout was associated with 3.49-fold higher adjusted odds of being in a higher reduced-hours-intention category (aOR = 3.49, 95% CI 2.16–5.66; *p* < 0.001).

## 4. Discussion

### 4.1. Summary of Findings

This study examined associations between occupational violence, organisational conditions, burnout and workforce withdrawal intentions among inpatient mental health staff. Occupational violence was highly prevalent, with verbal aggression reported by 93.9% of respondents and 41.7% experiencing it at least weekly. Burnout differed across verbal-aggression frequency categories in unadjusted analysis, although verbal aggression was not independently associated with burnout after adjustment. Lower perceived post-incident support and staffing adequacy were independently associated with higher burnout in the primary model, while higher burnout was strongly associated with greater intentions to leave the current role and reduce working hours.

### 4.2. Occupational Violence in Relation to Burnout

The high prevalence of occupational violence is consistent with previous studies demonstrating frequent aggression towards psychiatric staff [4,5]. Verbal aggression was almost universal, while threats, physical assault, sexually inappropriate behaviour and discriminatory abuse were also commonly reported. These findings reinforce occupational violence as a recurrent feature of inpatient mental health care rather than a series of isolated incidents.

Verbal aggression warrants particular attention, because repeated non-physical aggression may represent an important source of occupational strain. Previous studies have identified associations between workplace aggression or violence and burnout among healthcare staff [8,9]. In the present study, burnout differed across verbal-aggression categories and was weakly correlated with verbal-aggression frequency. However, the pattern was not consistently progressive and should not be interpreted as a dose–response relationship. Moreover, verbal aggression was no longer independently associated with burnout after adjustment.

This attenuation does not establish that organisational conditions mediate or explain the relationship between aggression and burnout. Instead, violence exposure and organisational conditions may represent overlapping aspects of the working environment. This is compatible with the Job Demands–Resources model, in which adverse occupational outcomes may arise when sustained job demands are insufficiently balanced by workplace resources [14,17]. Within mental health nursing, Park et al. similarly identified relationships between violence exposure, social support, the nursing practice environment and burnout [18].

The direction of these relationships cannot be determined from cross-sectional data. Staff exposed to frequent aggression may work in environments perceived as inadequately staffed or supported, while greater burnout may itself influence perceptions of organisational conditions. Prospective studies are required to establish temporal relationships.

### 4.3. Organisational Factors and Burnout

Lower perceived post-incident support and staffing adequacy were independently associated with higher burnout in the primary model. These findings are consistent with previous evidence linking psychiatric nursing work environments and organisational support with burnout and occupational outcomes [13,15,16].

Hierarchical analysis showed that the organisational variables contributed additional explained variance beyond staff characteristics and occupational violence exposures, whereas addition of the violence variables did not significantly improve model fit. This should not be interpreted as demonstrating that organisational factors are inherently more important than occupational violence: the contribution of sequential blocks depends partly on the variables included and their order of entry, and the observational design cannot establish causal priority. Rather, the organisational conditions measured accounted for additional variation in burnout within this sample.

Robustness analyses provided further context. Post-incident support remained negatively associated with burnout across all leave-one-out models and after inclusion of physical safety and training adequacy. Staffing adequacy also remained negatively associated throughout the leave-one-out analyses, although its association was attenuated in the expanded organisational model. Neither physical safety nor training adequacy was independently associated with burnout. The evidence for post-incident support was therefore particularly consistent, while the independent association with staffing adequacy was less certain when additional organisational conditions were considered simultaneously.

These findings align with Vincent-Höper et al., who identified follow-up counselling as potentially relevant to the relationship between workplace aggression and burnout, as well as with wider evidence linking modifiable workplace conditions and organisational culture with healthcare-worker mental health [8,20]. The authors extend this organisational perspective to multidisciplinary inpatient mental health staff, though their work remains observational in nature.

### 4.4. Implications of Burnout for Workforce Withdrawal Intentions

Higher burnout was strongly associated with greater intentions to leave the current role and reduce working hours, after adjustment for professional role, psychiatry experience and work pattern. This is consistent with previous evidence linking burnout with workforce turnover and other occupational consequences [11,12,21].

The association with intention to reduce working hours is also relevant because workforce withdrawal may occur without staff leaving their roles altogether, potentially affecting workforce capacity within already pressured inpatient services. However, these outcomes represent intentions rather than subsequent employment behaviour, and the cross-sectional design cannot establish that burnout causes workforce withdrawal. Longitudinal studies linking staff wellbeing with subsequent employment outcomes are therefore needed.

### 4.5. Implications for Practice and Future Research

The findings support consideration of staff wellbeing within a broader organisational framework alongside violence prevention. Strategies addressing occupational violence should recognise frequent verbal and other non-physical aggression in addition to physical assault. Perceived post-incident support was consistently associated with burnout across the primary and sensitivity analyses, while staffing adequacy was associated with burnout in the primary model but was less robust when additional organisational factors were considered.

These organisational conditions represent plausible areas for further service-level investigation, but the present study cannot establish that modifying them would reduce burnout or improve retention. Future longitudinal studies should examine whether occupational violence and organisational conditions predict subsequent changes in burnout and actual employment outcomes. Interventional research could evaluate whether strengthened post-incident support or improvements in staffing conditions are followed by improvements in staff wellbeing or retention. Multicentre studies would additionally help establish whether these associations are replicated across different inpatient settings and healthcare organisations.

### 4.6. Strengths and Limitations

A key strength of this study was the concurrent examination of occupational violence and organisational conditions among multidisciplinary inpatient mental health staff. The work-related burnout measure demonstrated excellent internal consistency (Cronbach’s α = 0.915; Supplementary Table S1). Additional strengths included regression diagnostics, robust standard-error estimation, influence and leave-one-out analyses, sensitivity analyses and ordinal regression appropriate to the ordered workforce withdrawal outcomes.

Several limitations should be considered. First, the cross-sectional design precludes causal or temporal inference [22]. Reverse causality cannot be excluded: staff experiencing greater burnout may perceive organisational conditions more negatively, while staff already considering workforce withdrawal may also report greater burnout. The observed associations therefore do not demonstrate that poorer organisational conditions caused burnout or that burnout caused subsequent workforce withdrawal.

Second, all data were self-reported and susceptible to recall and response bias, while measurement of exposures and outcomes within the same questionnaire introduces potential common-method bias [23]. Occupational violence, organisational conditions and workforce withdrawal intentions were assessed using study-specific measures that had not undergone formal psychometric validation, including several single-item measures, potentially reducing measurement precision and comparability with studies using validated instruments.

Third, the study was conducted within a single UK mental health trust and predominantly involved staff reporting St George’s Hospital as their primary site, with only three respondents reporting Redwoods Centre. Voluntary email and QR-code recruitment may have introduced self-selection bias, and a conventional response rate could not be calculated because the number of eligible staff receiving or viewing the invitation was unavailable. No a priori representative sample-size calculation was undertaken. Consequently, the sample should not be assumed to represent the wider NHS mental health workforce.

The anonymous questionnaire also collected a limited range of staff characteristics. Age, sex/gender, clinical grade, typical weekly hours, night-shift frequency and detailed shift patterns were unavailable, and residual confounding from factors such as objective workload, ward acuity, staffing ratios and restrictive-intervention frequency remains possible.

Four participants (3.5%) were excluded from the primary complete-case model because of missing post-incident support data. No significant differences were identified between included and excluded participants across available characteristics, although comparisons were limited by the small number excluded. The primary model also included several predictors relative to the analytical sample of 111. Although diagnostics and robustness analyses indicated generally stable findings, the modest sample may have reduced precision for smaller associations and limited examination of interactions and subgroup effects, consistent with limitations of smaller observational studies [24].

Finally, the prevalence of occupational violence should be interpreted according to the questionnaire definitions and six-week reporting period. In particular, sexually inappropriate or sexually harassing behaviour encompassed patient behaviours including sexual comments, gestures and unwanted exposure. The reported prevalence should therefore not be interpreted as representing only severe sexual harassment or assault.

## 5. Conclusions

Occupational violence was highly prevalent among inpatient mental health staff. Verbal aggression was associated with burnout in unadjusted analyses but not after adjustment, whereas lower perceived post-incident support and staffing adequacy were associated with higher burnout in the primary model. The staffing association was attenuated when additional organisational factors were considered. Higher burnout was also strongly associated with greater intentions to leave the current role and reduce working hours.

These findings identify organisational conditions that warrant further investigation alongside occupational violence prevention but do not establish causal relationships. Larger longitudinal, multicentre and interventional studies are needed to determine whether changes in organisational conditions are followed by reductions in burnout and improved workforce retention.

## Figures and Tables

**Figure 1:**
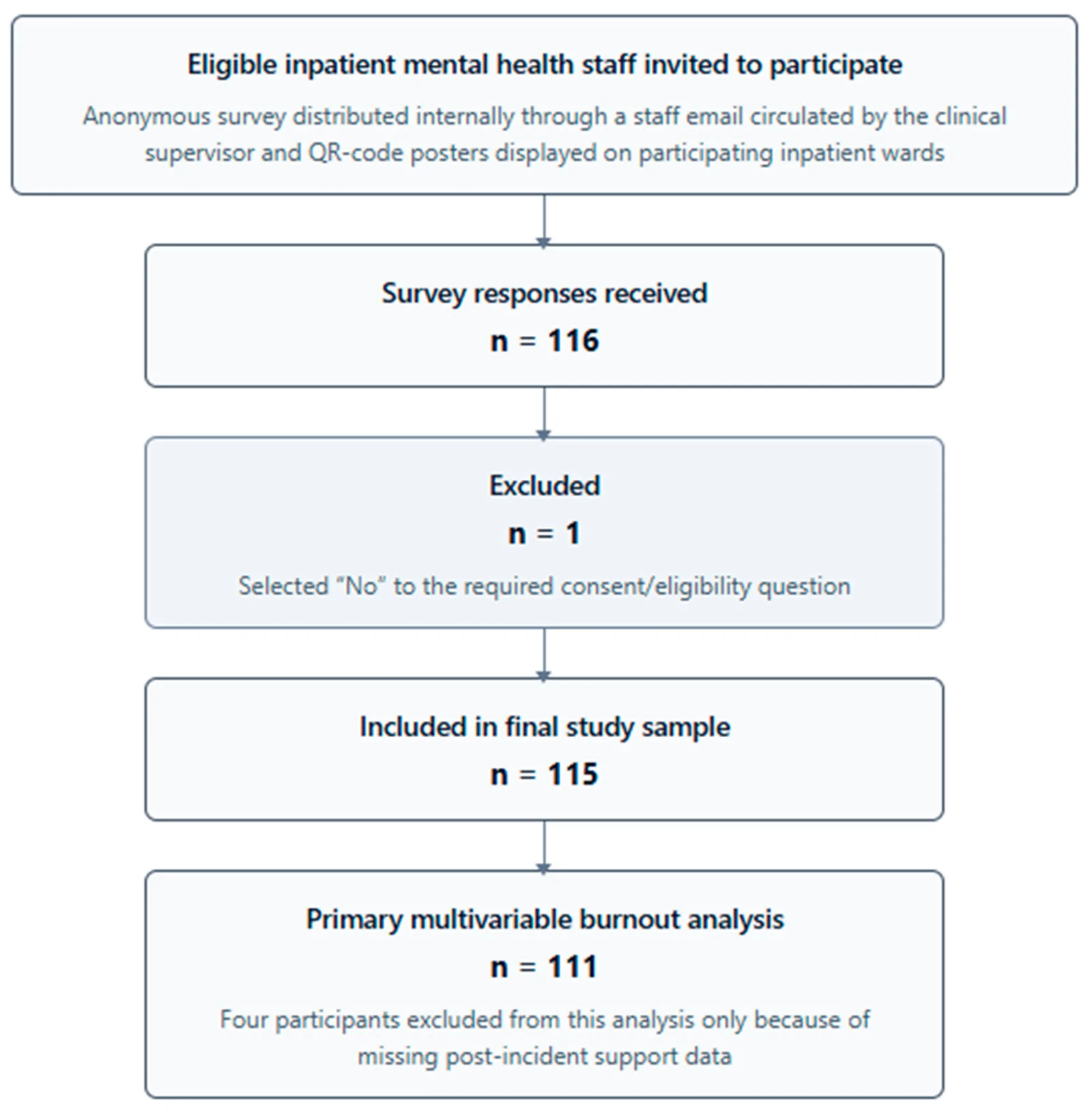
Participant recruitment and inclusion flowchart.

**Table 1:** Participant characteristics, occupational violence exposure, organisational perceptions, and burnout scores among inpatient mental health staff (*n* = 115).

**Primary site of work**	
St George’s Hospital, *n* (%)	112 (97.4%)
Redwoods, *n* (%)	3 (2.6%)
**Work setting**	
Adult acute inpatient ward, *n* (%)	45 (39.1%)
Psychiatric intensive care unit (PICU), *n* (%)	10 (8.7%)
Older adult inpatient ward, *n* (%)	16 (13.9%)
Forensic ward, *n* (%)	30 (26.1%)
Other, *n* (%)	14 (12.2%)
**Professional role**	
Consultant psychiatrist, *n* (%)	6 (5.2%)
Core trainee/specialty doctor, *n* (%)	9 (7.8%)
Foundation doctor, *n* (%)	6 (5.2%)
Registered mental health nurse, *n* (%)	39 (33.9%)
Healthcare assistant/support worker, *n* (%)	27 (23.5%)
Psychologist, *n* (%)	4 (3.5%)
Physiotherapist/occupational therapist, *n* (%)	8 (7.0%)
Other, *n* (%)	16 (13.9%)
**Years working in psychiatry**	
<6 months, *n* (%)	11 (9.6%)
6–12 months, *n* (%)	6 (5.2%)
1–3 years, *n* (%)	22 (19.1%)
3–5 years, *n* (%)	25 (21.7%)
>5 years, *n* (%)	51 (44.3%)
**Work pattern**	
Full-time, *n* (%)	97 (84.3%)
Less than full-time, *n* (%)	18 (15.7%)
**Occupational violence exposure in previous 6 weeks**	
Any verbal aggression, *n* (%)	108 (93.9%)
Verbal aggression weekly or more often, *n* (%)	48 (41.7%)
Any threats of physical harm, *n* (%)	85 (73.9%)
Any physical assault, *n* (%)	67 (58.3%)
Any sexually inappropriate or sexually harassing behaviour from patients, *n* (%)	83 (72.2%)
Any discriminatory/racist abuse, *n* (%)	66 (57.4%)
**Organisational variables**	
Staffing adequacy, mean (SD)	2.29 (1.09)
Post-incident support, mean (SD) *	2.64 (0.99)
Physical safety at work, mean (SD)	2.68 (0.92)
Training adequacy, mean (SD)	2.70 (0.95)
**Burnout score, mean (SD)**	1.92 (0.87)

Occupational violence exposures refer to incidents reported during the preceding six weeks. Organisational variables were measured using five-point Likert scales ranging from “Strongly disagree” to “Strongly agree”, with higher scores indicating more favourable organisational perceptions. Burnout was measured using a seven-item composite scale derived from the Copenhagen Burnout Inventory work-related burnout subscale, with higher scores indicating greater burnout. * Post-incident support data were available for 111 participants; all other organisational variables and the burnout composite were available for all 115 participants.

**Table 2:** Multivariable linear regression analysis of factors associated with burnout among inpatient mental health staff.

Predictor	B (95% CI)	Standardised β	*p*-Value
Verbal aggression	0.116 (−0.058 to 0.290)	0.17	0.191
Threats of physical harm	0.026 (−0.174 to 0.226)	0.04	0.798
Physical assault	−0.105 (−0.338 to 0.128)	−0.11	0.374
Staffing adequacy	−0.213 (−0.383 to −0.042)	−0.27	0.015
Post-incident support	−0.278 (−0.442 to −0.113)	−0.32	0.001
Professional role: Nursing vs. Medical	−0.206 (−0.688 to 0.276)	—	0.398
Professional role: Support/HCA vs. Medical	−0.375 (−0.909 to 0.159)	—	0.167
Professional role: AHP/Other vs. Medical	−0.314 (−0.798 to 0.170)	—	0.201
Years working in psychiatry	−0.014 (−0.145 to 0.117)	−0.02	0.833
Work pattern	−0.038 (−0.455 to 0.379)	−0.02	0.856

Higher burnout scores indicate greater work-related burnout. All variables shown were entered simultaneously. Medical staff were the reference category for professional role. B represents the unstandardised regression coefficient; β represents the standardised regression coefficient; CI, confidence interval; HCA, healthcare assistant; AHP, allied health professional. Overall model: *F*(10,100) = 3.72, *p* < 0.001; R^2^ = 0.271; adjusted R^2^ = 0.198.

**Table 3:** Associations between burnout and workforce withdrawal intentions among inpatient mental health staff.

Outcome	Predictor	aOR (95% CI, *p*-Value)	Model
Intention to leave current role	Burnout score	11.54 (5.96–22.34, <0.001)	Partial proportional odds
Intention to reduce working hours	Burnout score	3.49 (2.16–5.66, <0.001)	Proportional odds

Models were adjusted for professional role group, years working in psychiatry, and work pattern. Adjusted odds ratios (aORs) represent the change in odds of being in a higher intention category per one-unit increase in burnout score. A partial proportional-odds model was used for intention to leave because the proportional-odds assumption was not satisfied for years working in psychiatry (*p* = 0.044); a proportional-odds model was used for intention to reduce working hours. CI, confidence interval; aOR, adjusted odds ratio.

## Data Availability

The datasets generated and/or analysed during the current study are not publicly available because they contain staff survey responses from a single NHS mental health trust and public release could risk deductive disclosure; they are available from the corresponding author on reasonable request.

## Supplementary Materials

The following supporting information can be downloaded at: https://www.mdpi.com/article/10.3390/healthcare14193158/s1, Table S1: Psychometric properties of the burnout composite scale; Table S2: Frequency distributions of occupational violence exposure and seclusion involvement among inpatient mental health staff; Table S3: Burnout scores by verbal aggression frequency category; Table S4: Correlation matrix of burnout, occupational violence exposures, organisational factors, and workforce withdrawal intentions; Table S5: Hierarchical regression analysis of factors associated with burnout; Table S6: Burnout and occupational violence exposure stratified by professional role group; Figure S1: Mean burnout scores according to verbal aggression frequency; Figure S2: Multivariable associations with burnout among inpatient mental health staff; Figure S3: Distribution of burnout scores among inpatient mental health staff.
