# Supplementary material for: Occupational Violence, Organisational Factors, Burnout and Workforce Withdrawal Intentions Among Inpatient Mental Health Staff: A Cross-Sectional Survey
Source: Healthcare (Basel). 2026 Sep 23;14(19):3158. doi: 10.3390/healthcare14193158 (PMC13665279; doi:10.3390/healthcare14193158)
Supplement: Supplementary file 1 [file healthcare-14-03158-s001.zip › healthcare-4502158-supplementary.pdf]

Supplementary material for: Occupational Violence, Organisational Factors, Burnout and Workforce Withdrawal Intentions Among Inpatient Mental Health Staff: A Cross-Sectional Survey

This supplementary appendix contains additional descriptive analyses, psychometric analyses, subgroup analyses, and supporting figures referenced in the main manuscript.

Supplementary Table S1. Psychometric properties of the burnout composite scale.

Burnout item	Corrected item–total correlation	Cronbach's α if item removed
Worn out at end of working day	0.77	0.90
Exhausted in the morning at thought of work	0.84	0.89
Every working hour is tiring	0.81	0.89
Enough energy for family/friends	0.51	0.92
Work emotionally exhausting	0.67	0.91
Work frustrating	0.72	0.90
Burnt out because of work	0.85	0.89

Psychometric properties of the seven-item burnout composite scale. Corrected item–total correlations and Cronbach's alpha if each item was removed are presented. Overall internal consistency of the seven-item composite was high (Cronbach's $\alpha = 0.915$). The item assessing energy for family and friends was reverse scored so that higher scores consistently represented greater work-related burnout.

Supplementary Table S2. Frequency distributions of occupational violence exposure and seclusion involvement among inpatient mental health staff.

**Exposure frequency**	**Never**	**Once**	**Few times**	**Weekly**	**Several times/week**	**Daily/ almost daily**
Verbal aggression, n (%)	7 (6.1%)	8 (7.0%)	52 (45.2%)	17 (14.8%)	21 (18.3%)	10 (8.7%)
Threats of physical harm, n (%)	30 (26.1%)	3 (2.6%)	62 (53.9%)	11 (9.6%)	6 (5.2%)	3 (2.6%)
Attempted physical assault, n (%)	32 (27.8%)	7 (6.1%)	61 (53.0%)	7 (6.1%)	6 (5.2%)	2 (1.7%)
Physical assault, n (%)	48 (41.7%)	20 (17.4%)	46 (40.0%)	1 (0.9%)	—	—
Sexual harassment/inappropriate behaviour, n (%)	32 (27.8%)	12 (10.4%)	56 (48.7%)	9 (7.8%)	2 (1.7%)	4 (3.5%)
Discriminatory/racist abuse, n (%)	49 (42.6%)	11 (9.6%)	49 (42.6%)	2 (1.7%)	2 (1.7%)	2 (1.7%)
Seclusion involvement, n (%)	36 (31.3%)	14 (12.2%)	49 (42.6%)	8 (7.0%)	6 (5.2%)	2 (1.7%)

Detailed frequency distributions of occupational violence exposure and seclusion involvement among inpatient mental health staff. Exposure frequencies were assessed using Likert-scale response categories during the preceding six weeks.

Supplementary Table S3. Burnout scores by verbal aggression frequency category

Verbal aggression frequency	n	Mean burnout	SD
**Never**	7	1.14	0.64
**Once**	8	2.11	0.69
**Few times**	52	1.86	0.95
**Weekly**	17	2.06	0.78
**Several times/week**	21	1.85	0.73
**Daily/almost daily**	10	2.51	0.83

One-way ANOVA: $F(5,109) = 2.43$, $p = 0.040$, $\eta^2 = 0.10$. Levene's test indicated no evidence of unequal variances ($p = 0.370$). Tukey-adjusted post-hoc comparisons identified a significant difference only between the "Daily/almost daily" and "Never" groups (mean difference = 1.37, adjusted $p = 0.017$). Welch ANOVA: $F(5,27.27) = 3.09$, $p = 0.025$.

Supplementary Table S4. Correlation matrix of burnout, occupational violence exposures, organisational factors, and workforce withdrawal intentions.

Variable	1	2	3	4	5	6	7	8
**1. Burnout**	1.00							
**2. Verbal aggression**	0.20*	1.00						
**3. Threats**	0.16	0.74***	1.00					
**4. Physical assault**	0.06	0.56***	0.66***	1.00				
**5. Staffing adequacy**	−0.42***	−0.27**	−0.29**	−0.21*	1.00			
**6. Post-incident support**	−0.41***	0.07	0.10	−0.02	0.40***	1.00		
**7. Turnover intention**	0.66***	0.12	0.09	0.04	−0.46***	−0.40***	1.00	

**8. Reduce-hours intention**	0.45***	0.03	0.02	0.02	−0.29**	−0.36***	0.62***	1.00

Spearman correlation matrix between burnout, occupational violence exposures, organisational variables and workforce withdrawal intentions. Values are Spearman's rank correlation coefficients (ρ). Pairwise complete observations were used; $n = 115$ for correlations not involving post-incident support and $n = 111$ for correlations involving post-incident support. * $p < 0.05$; ** $p < 0.01$; *** $p < 0.001$.

Supplementary Table S5. Hierarchical regression analysis of factors associated with burnout.

Step	**Variables entered**	R^2	**Adjusted R^2**	ΔR^2	**F-change**	**p-value**
1	Professional role group, years working in psychiatry, work pattern	0.025	−0.021	—	—	—
2	+ Verbal aggression, threats of physical harm, physical assault	0.062	−0.012	0.036	1.67	0.179
3	+ Staffing adequacy, post-incident support	0.271	0.198	0.209	14.35	<0.001

Hierarchical multivariable linear regression examining incremental variance in burnout explained by staff-level characteristics, occupational violence exposures and organisational factors (n = 111). Step 1 included professional role group, years working in

psychiatry and work pattern. Step 2 added verbal aggression, threats of physical harm and physical assault. Step 3 added perceived staffing adequacy and post-incident support. The ordering reflected the conceptual framework of the study, distinguishing staff-level characteristics from occupational demands and potentially modifiable organisational conditions, and was not determined by statistical significance. ΔR^2 represents the additional proportion of variance explained relative to the preceding model. F-change and *p*-values compare each model with the preceding step.

Supplementary Table S6. Burnout and occupational violence exposure stratified by professional role group.

**Role group**	**n**	**Burnout mean (SD)**	**Verbal aggression mean (SD)**
Medical staff	21	1.92 (1.09)	2.14 (1.11)
Nursing staff	39	1.97 (0.76)	2.87 (1.28)
Support/HCA staff	27	1.99 (0.88)	2.93 (1.36)
AHP/other staff	28	1.76 (0.86)	2.18 (1.19)

Burnout and occupational violence exposure stratified by professional role group. Comparisons between professional role groups were performed using one-way analysis of variance.

Supplementary Figure S1. Mean burnout scores according to verbal aggression frequency.

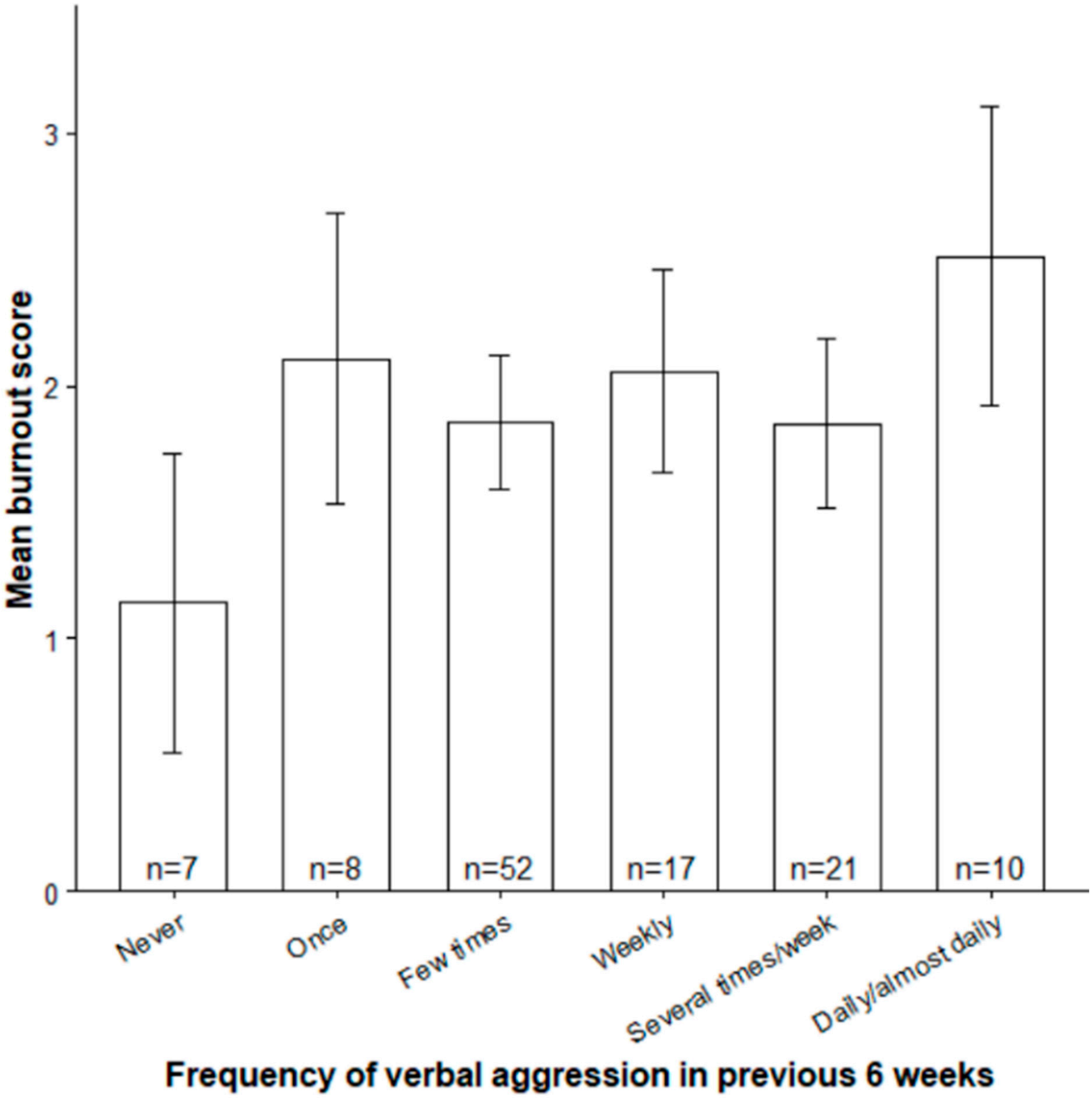

Mean burnout scores according to verbal aggression frequency among inpatient mental health staff. Bars represent mean burnout scores across verbal aggression exposure frequency categories during the preceding six weeks. Error bars represent 95% confidence intervals.

Supplementary Figure S2. Multivariable associations with burnout among inpatient mental health staff.

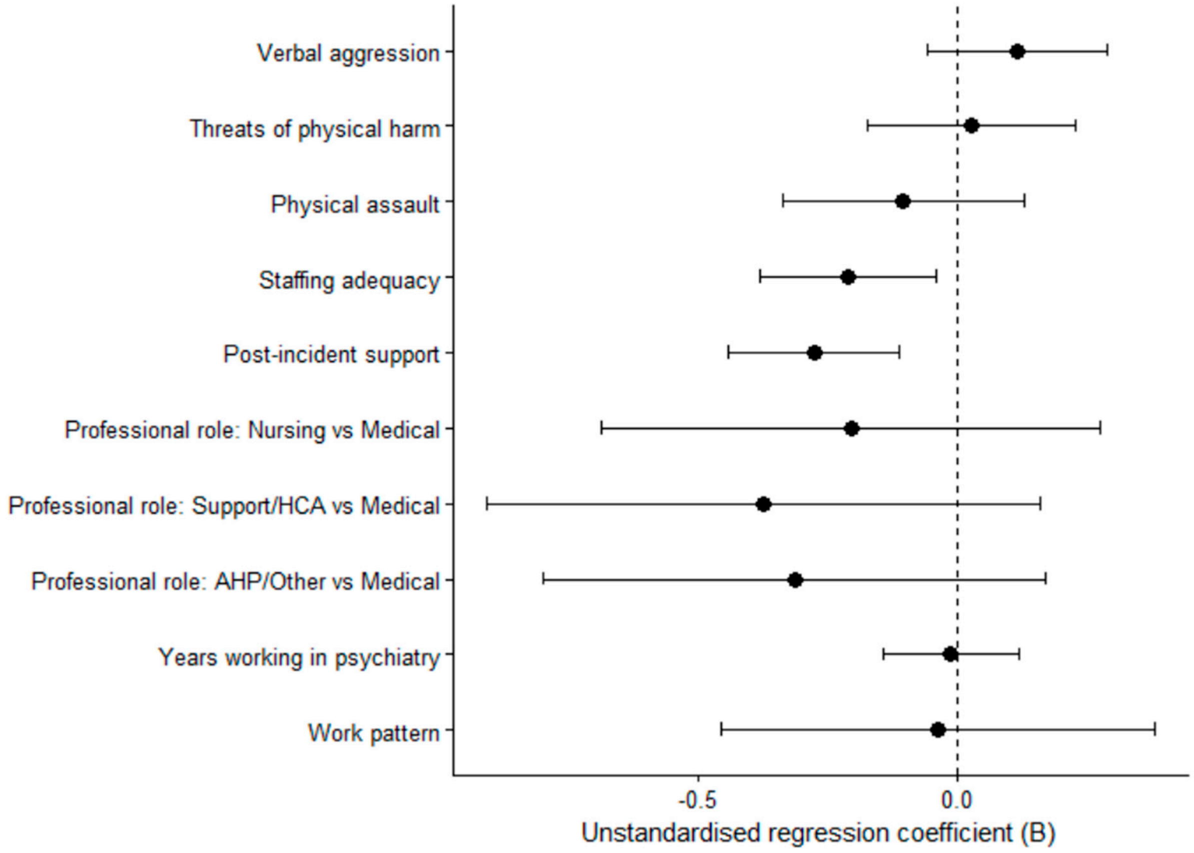

Forest plot showing unstandardised regression coefficients (B) and 95% confidence intervals from the primary multivariable linear regression model (n = 111). The vertical dashed line indicates the null value (B = 0). Negative coefficients indicate associations with lower burnout scores. Medical staff were the reference category for professional role.

Supplementary Figure S3. Distribution of burnout scores among inpatient mental health staff.

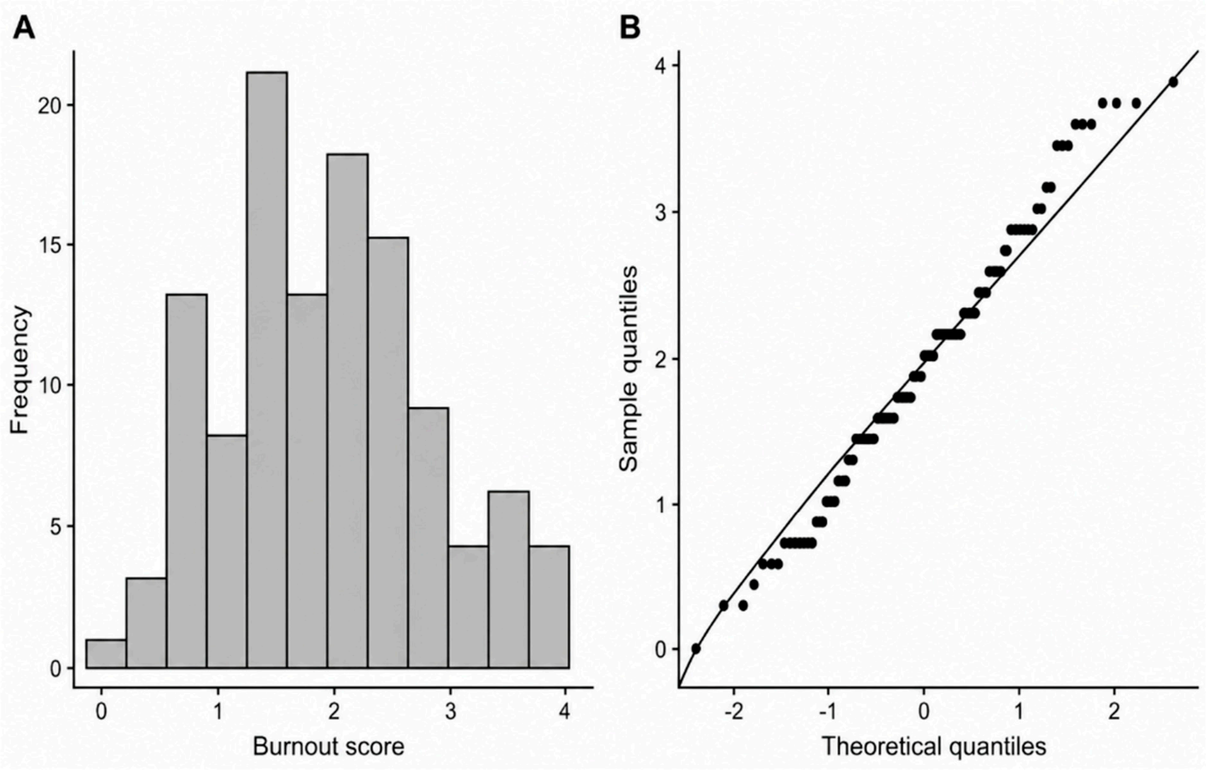

Distribution of burnout scores among inpatient mental health staff.

(A) Histogram demonstrating distribution of burnout scores.

(B) Quantile–quantile plot assessing normality of burnout scores.
